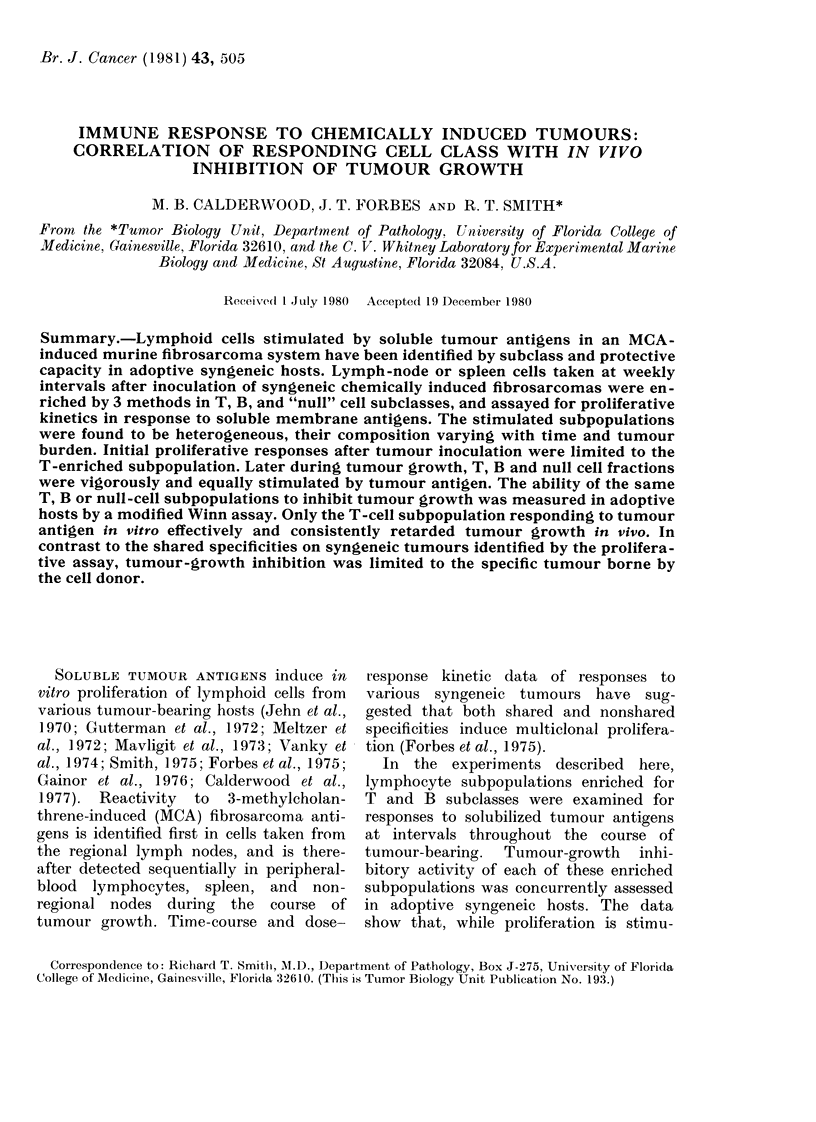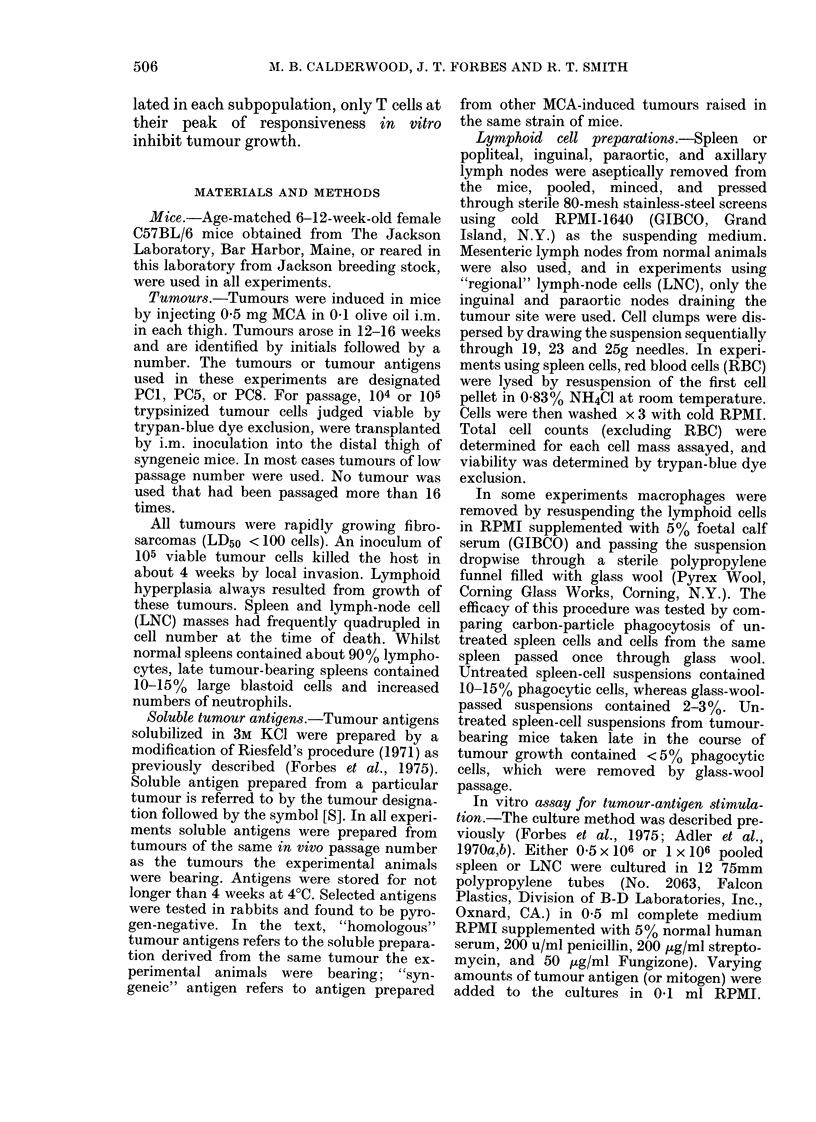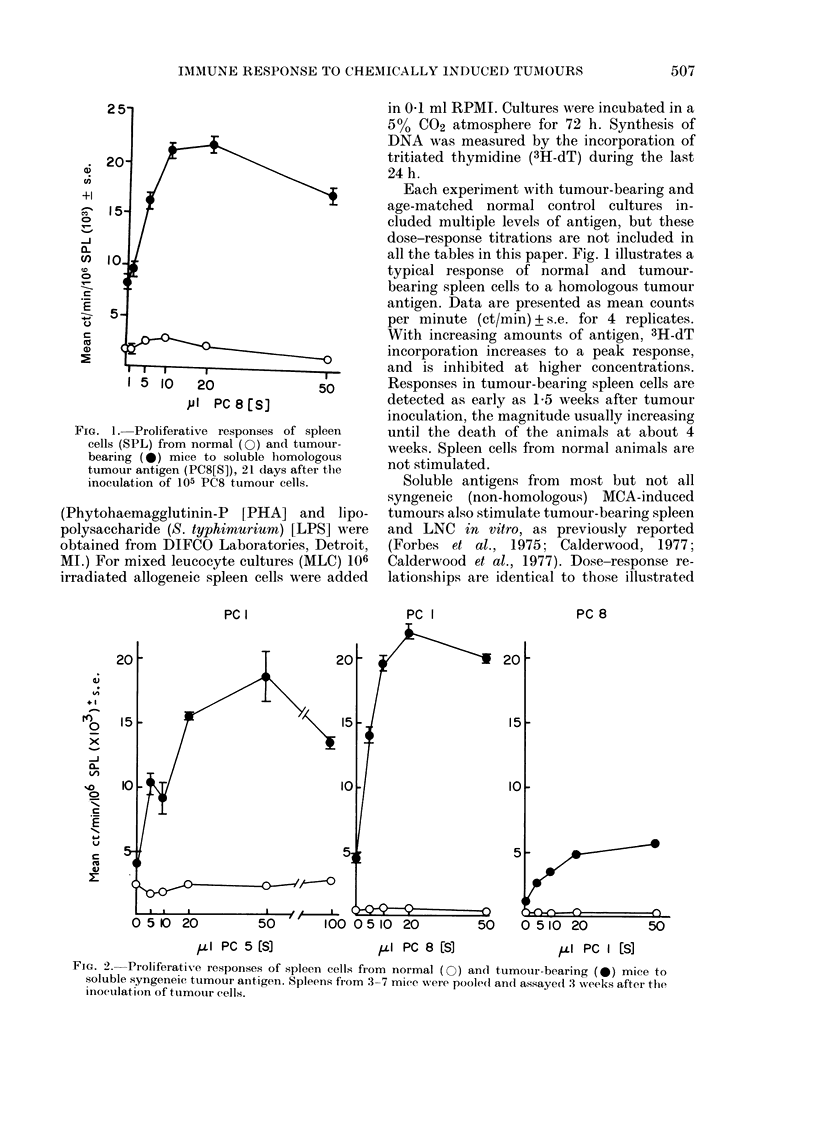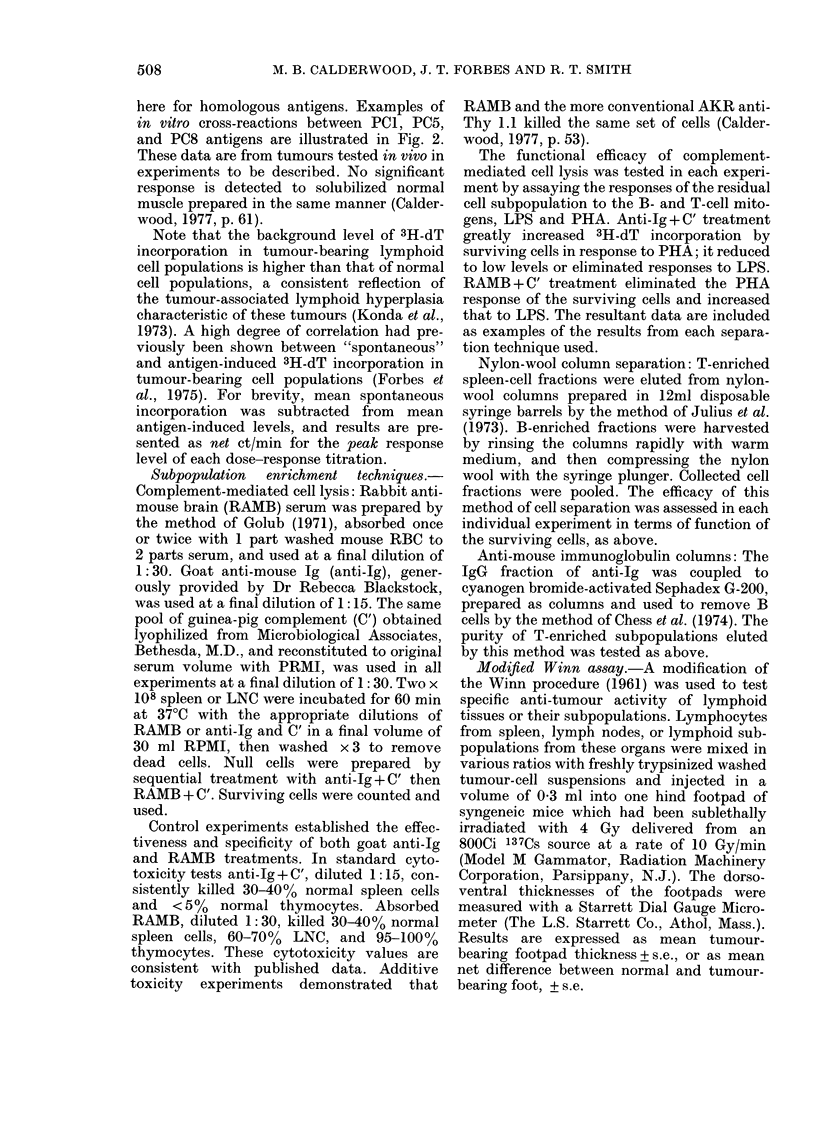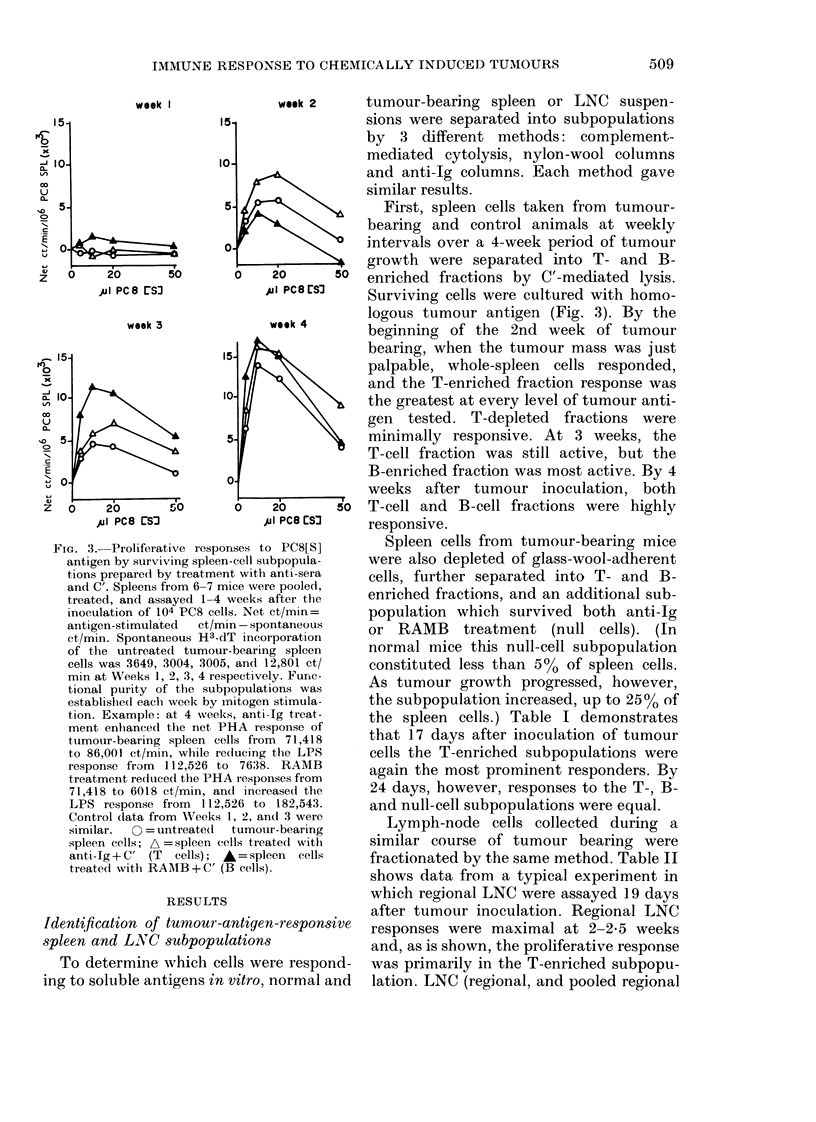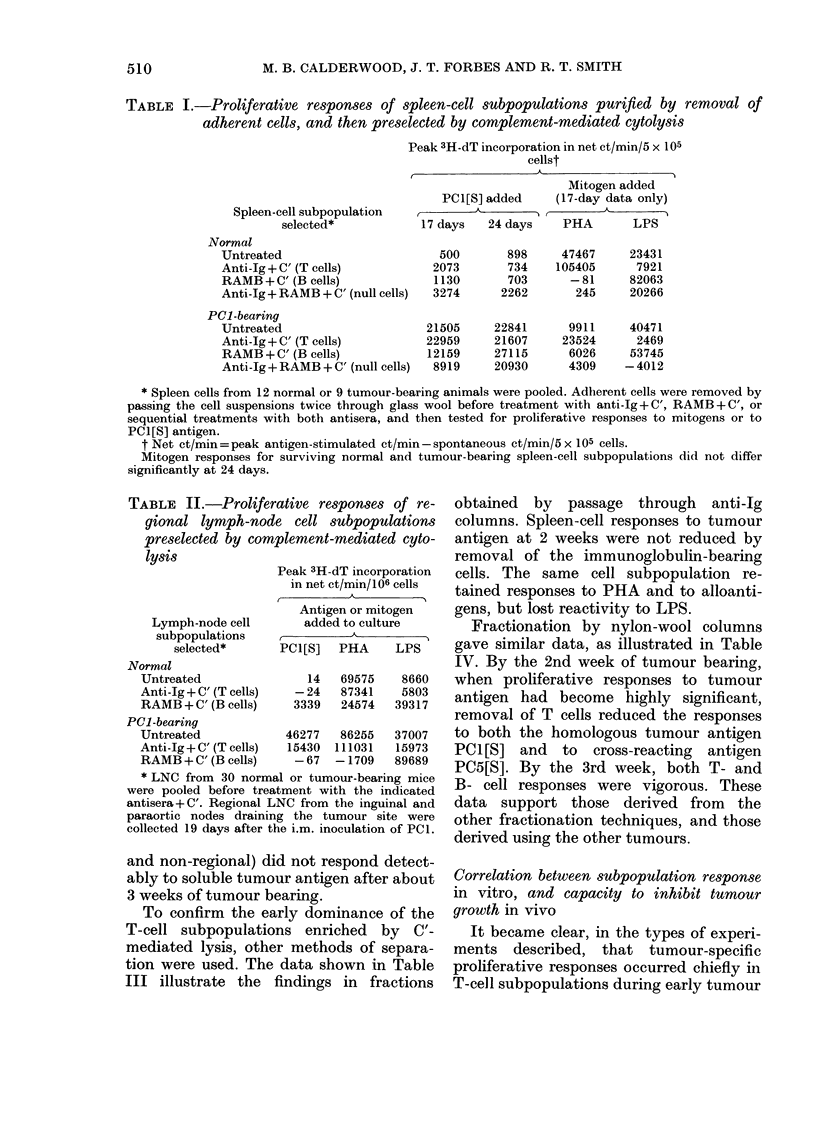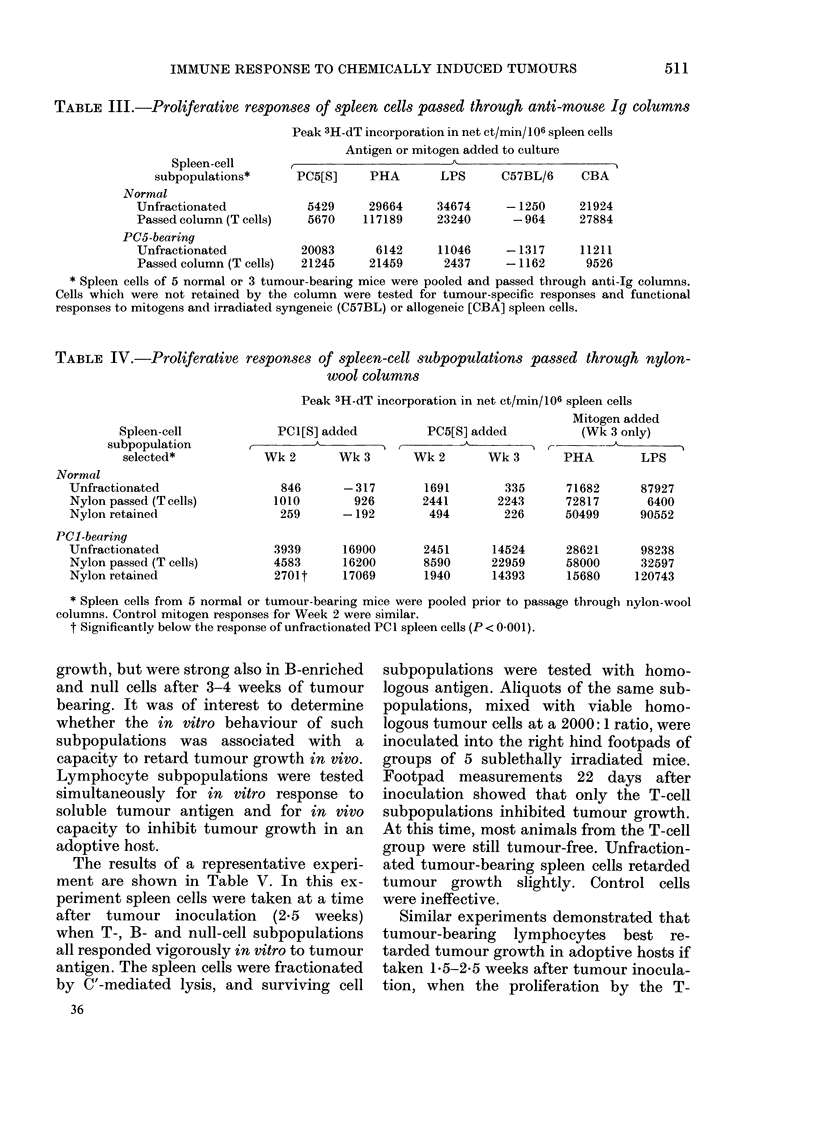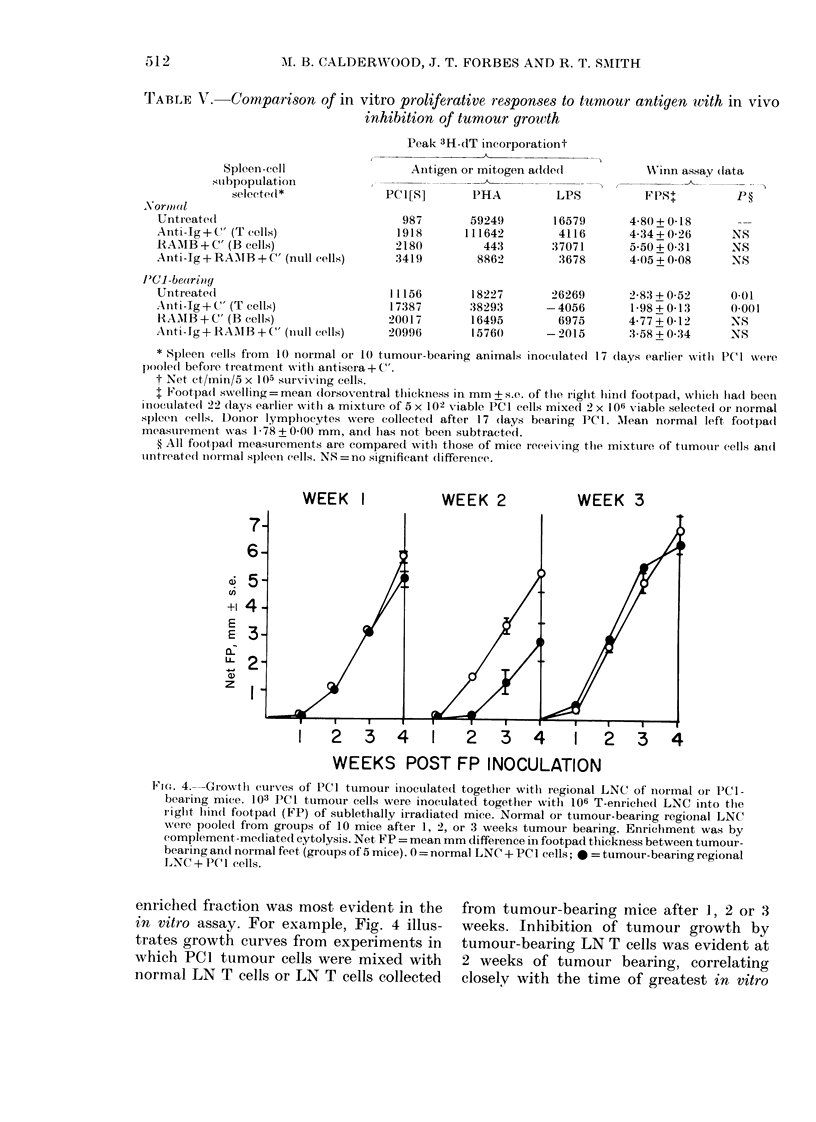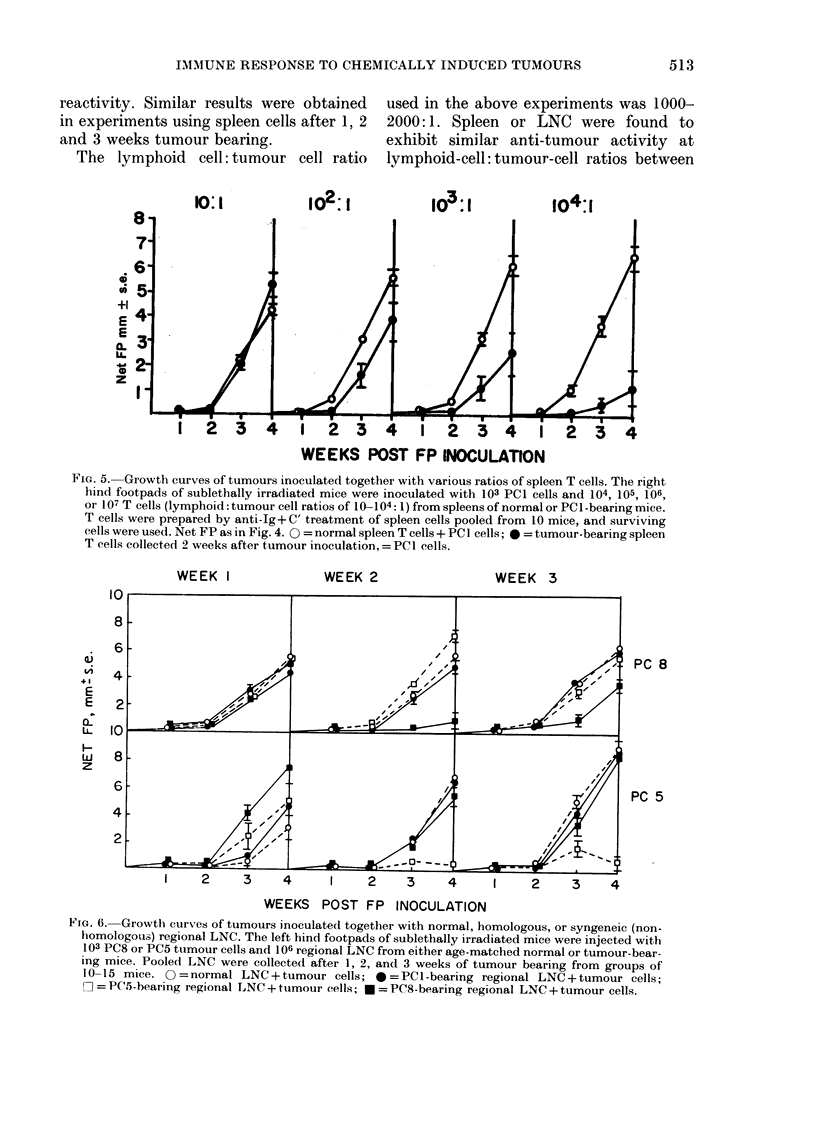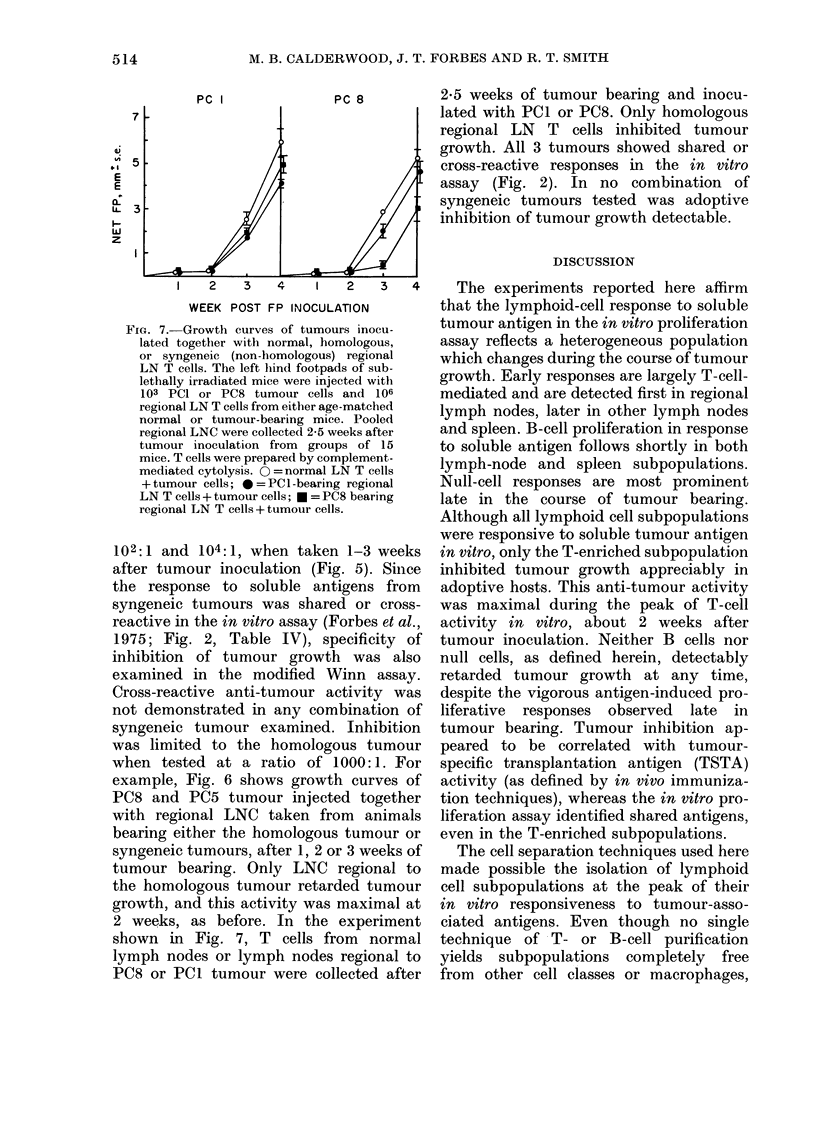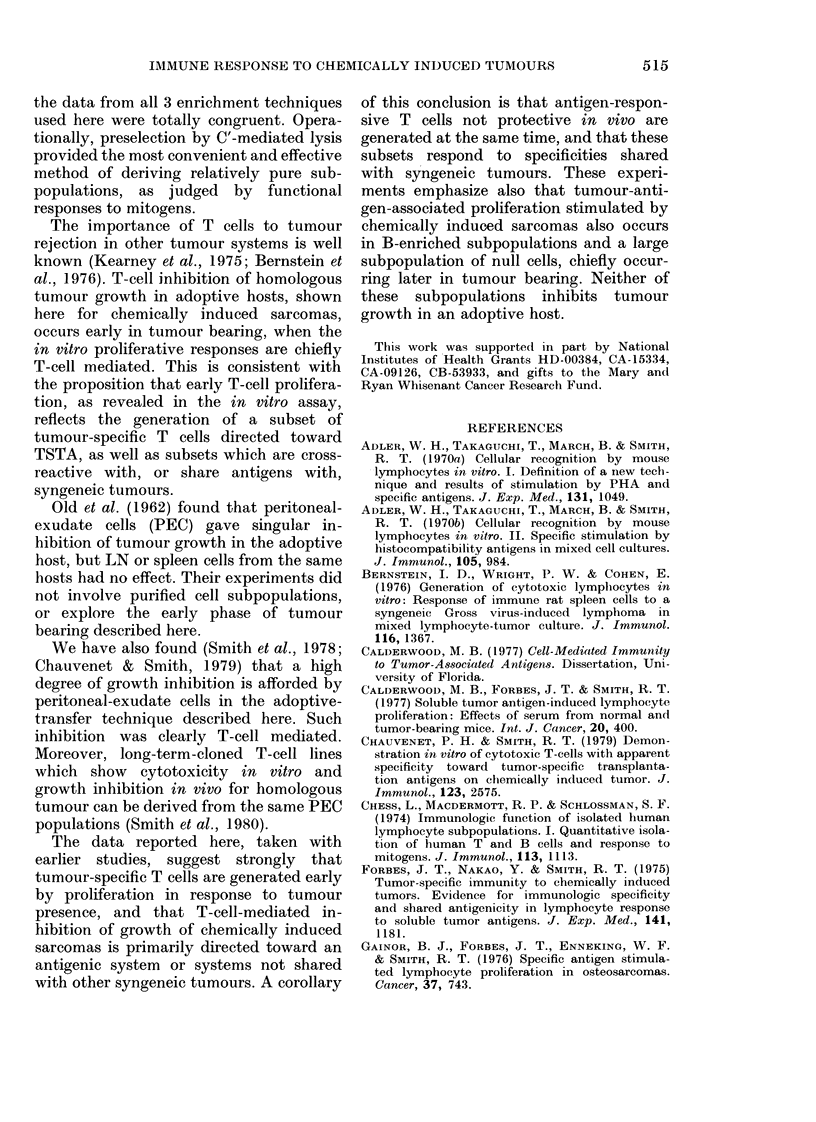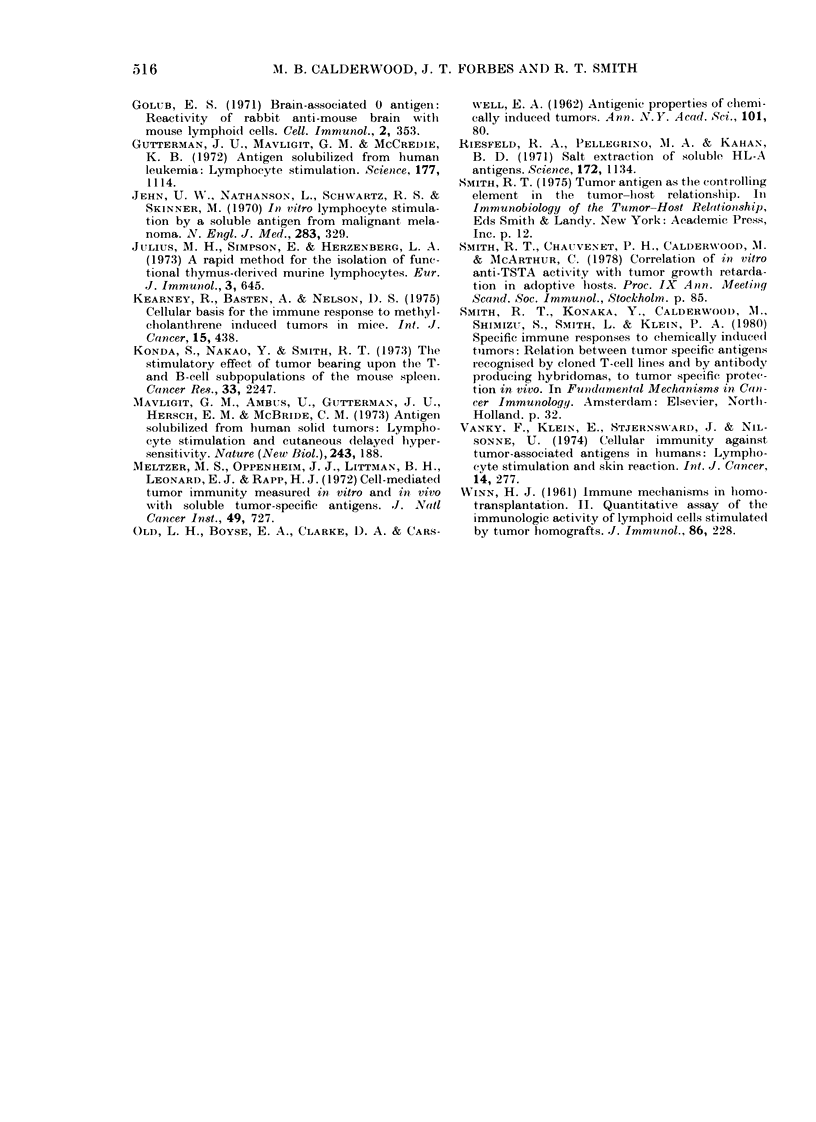# Immune response to chemically induced tumours: correlation of responding cell class with in vivo inhibition of tumour growth.

**DOI:** 10.1038/bjc.1981.73

**Published:** 1981-04

**Authors:** M. B. Calderwood, J. T. Forbes, R. T. Smith

## Abstract

Lymphoid cells stimulated by soluble tumour antigens in the MCA-induced murine fibrosarcoma system have been identified by subclass and protective capacity in adoptive syngeneic hosts. Lymph-node or spleen cells taken at weekly intervals after inoculation of syngeneic chemically induced fibrosarcomas were enriched by 3 methods in T, B, and "null" cell subclasses, and assayed for proliferative kinetics in response to soluble membrane antigens. The stimulated subpopulations were found to be heterogeneous, their composition varying with time and tumour burden. Initial proliferative responses after tumour inoculation were limited to the T-enriched subpopulation. Later during tumour growth, T, B and null cell fractions were vigorously and equally stimulated by tumour antigen. The ability of the same T, B or null-cell subpopulations to inhibit tumour growth was measured in adoptive hosts by a modified Winn assay. Only the T-cell subpopulation responding to tumour antigen in vitro effectively and consistently retarded tumour growth in vivo. In contrast to the shared specificities on syngeneic tumours identified by the proliferative assay, tumour-growth inhibition was limited to the specific tumour borne by the cell donor.


*Br. J. Cancer* (1981) **43**, 505

### IMMUNE RESPONSE TO CHEMICALLY INDUCED TUMOURS: CORRELATION OF RESPONDING CELL CLASS WITH *IN VIVO* INHIBITION OF TUMOUR GROWTH

M. B. CALDERWOOD, J. T. FORBES AND R. T. SMITH*

*From the *Tumor Biology Unit, Department of Pathology, University of Florida College of Medicine, Gainesville, Florida* 32610, *and the C. V. Whitney Laboratory for Experimental Marine Biology and Medicine, St Augustine, Florida* 32084, *U.S.A.*

Received 1 July 1980 Accepted 19 December 1980

**Summary.—Lymphoid cells stimulated by soluble tumour antigens in an MCA-induced murine fibrosarcoma system have been identified by subclass and protective capacity in adoptive syngeneic hosts. Lymph-node or spleen cells taken at weekly intervals after inoculation of syngeneic chemically induced fibrosarcomas were enriched by 3 methods in T, B, and "null" cell subclasses, and assayed for proliferative kinetics in response to soluble membrane antigens. The stimulated subpopulations were found to be heterogeneous, their composition varying with time and tumour burden. Initial proliferative responses after tumour inoculation were limited to the T-enriched subpopulation. Later during tumour growth, T, B and null cell fractions were vigorously and equally stimulated by tumour antigen. The ability of the same T, B or null-cell subpopulations to inhibit tumour growth was measured in adoptive hosts by a modified Winn assay. Only the T-cell subpopulation responding to tumour antigen *in vitro* effectively and consistently retarded tumour growth *in vivo*. In contrast to the shared specificities on syngeneic tumours identified by the proliferative assay, tumour-growth inhibition was limited to the specific tumour borne by the cell donor.**

SOLUBLE TUMOUR ANTIGENS induce *in vitro* proliferation of lymphoid cells from various tumour-bearing hosts (Jehn *et al.*, 1970; Gutterman *et al.*, 1972; Meltzer *et al.*, 1972; Mavligit *et al.*, 1973; Vanky *et al.*, 1974; Smith, 1975; Forbes *et al.*, 1975; Gainor *et al.*, 1976; Calderwood *et al.*, 1977). Reactivity to 3-methylcholanthrene-induced (MCA) fibrosarcoma antigens is identified first in cells taken from the regional lymph nodes, and is thereafter detected sequentially in peripheral-blood lymphocytes, spleen, and non-regional nodes during the course of tumour growth. Time-course and dose-response kinetic data of responses to various syngeneic tumours have suggested that both shared and nonshared specificities induce multiclonal proliferation (Forbes *et al.*, 1975).

In the experiments described here, lymphocyte subpopulations enriched for T and B subclasses were examined for responses to solubilized tumour antigens at intervals throughout the course of tumour-bearing. Tumour-growth inhibitory activity of each of these enriched subpopulations was concurrently assessed in adoptive syngeneic hosts. The data show that, while proliferation is stimu-

Correspondence to: Richard T. Smith, M.D., Department of Pathology, Box J-275, University of Florida College of Medicine, Gainesville, Florida 32610. (This is Tumor Biology Unit Publication No. 193.)

lated in each subpopulation, only T cells at their peak of responsiveness *in vitro* inhibit tumour growth.

### MATERIALS AND METHODS

*Mice*.—Age-matched 6–12-week-old female C57BL/6 mice obtained from The Jackson Laboratory, Bar Harbor, Maine, or reared in this laboratory from Jackson breeding stock, were used in all experiments.

*Tumours*.—Tumours were induced in mice by injecting 0·5 mg MCA in 0·1 olive oil i.m. in each thigh. Tumours arose in 12–16 weeks and are identified by initials followed by a number. The tumours or tumour antigens used in these experiments are designated PC1, PC5, or PC8. For passage, $10^4$ or $10^5$ trypsinized tumour cells judged viable by trypan-blue dye exclusion, were transplanted by i.m. inoculation into the distal thigh of syngeneic mice. In most cases tumours of low passage number were used. No tumour was used that had been passaged more than 16 times.

All tumours were rapidly growing fibrosarcomas ($LD_{50}$ <100 cells). An inoculum of $10^5$ viable tumour cells killed the host in about 4 weeks by local invasion. Lymphoid hyperplasia always resulted from growth of these tumours. Spleen and lymph-node cell (LNC) masses had frequently quadrupled in cell number at the time of death. Whilst normal spleens contained about 90% lymphocytes, late tumour-bearing spleens contained 10–15% large blastoid cells and increased numbers of neutrophils.

*Soluble tumour antigens*.—Tumour antigens solubilized in 3M KCl were prepared by a modification of Riesfeld's procedure (1971) as previously described (Forbes *et al*., 1975). Soluble antigen prepared from a particular tumour is referred to by the tumour designation followed by the symbol [S]. In all experiments soluble antigens were prepared from tumours of the same *in vivo* passage number as the tumours the experimental animals were bearing. Antigens were stored for not longer than 4 weeks at 4°C. Selected antigens were tested in rabbits and found to be pyrogen-negative. In the text, "homologous" tumour antigens refers to the soluble preparation derived from the same tumour the experimental animals were bearing; "syngeneic" antigen refers to antigen prepared from other MCA-induced tumours raised in the same strain of mice.

*Lymphoid cell preparations*.—Spleen or popliteal, inguinal, paraortic, and axillary lymph nodes were aseptically removed from the mice, pooled, minced, and pressed through sterile 80-mesh stainless-steel screens using cold RPMI-1640 (GIBCO, Grand Island, N.Y.) as the suspending medium. Mesenteric lymph nodes from normal animals were also used, and in experiments using "regional" lymph-node cells (LNC), only the inguinal and paraortic nodes draining the tumour site were used. Cell clumps were dispersed by drawing the suspension sequentially through 19, 23 and 25g needles. In experiments using spleen cells, red blood cells (RBC) were lysed by resuspension of the first cell pellet in 0·83% $NH_4Cl$ at room temperature. Cells were then washed ×3 with cold RPMI. Total cell counts (excluding RBC) were determined for each cell mass assayed, and viability was determined by trypan-blue dye exclusion.

In some experiments macrophages were removed by resuspending the lymphoid cells in RPMI supplemented with 5% foetal calf serum (GIBCO) and passing the suspension dropwise through a sterile polypropylene funnel filled with glass wool (Pyrex Wool, Corning Glass Works, Corning, N.Y.). The efficacy of this procedure was tested by comparing carbon-particle phagocytosis of untreated spleen cells and cells from the same spleen passed once through glass wool. Untreated spleen-cell suspensions contained 10–15% phagocytic cells, whereas glass-wool-passed suspensions contained 2–3%. Untreated spleen-cell suspensions from tumour-bearing mice taken late in the course of tumour growth contained <5% phagocytic cells, which were removed by glass-wool passage.

In vitro *assay for tumour-antigen stimulation*.—The culture method was described previously (Forbes *et al*., 1975; Adler *et al*., 1970*a*,*b*). Either $0·5 \times 10^6$ or $1 \times 10^6$ pooled spleen or LNC were cultured in 12 75mm polypropylene tubes (No. 2063, Falcon Plastics, Division of B-D Laboratories, Inc., Oxnard, CA.) in 0·5 ml complete medium RPMI supplemented with 5% normal human serum, 200 u/ml penicillin, 200 μg/ml streptomycin, and 50 μg/ml Fungizone). Varying amounts of tumour antigen (or mitogen) were added to the cultures in 0·1 ml RPMI.

FIG. 1.—Proliferative responses of spleen cells (SPL) from normal (○) and tumour-bearing (●) mice to soluble homologous tumour antigen (PC8[S]), 21 days after the inoculation of $10^5$ PC8 tumour cells.

(Phytohaemagglutinin-P [PHA] and lipopolysaccharide (*S. typhimurium*) [LPS] were obtained from DIFCO Laboratories, Detroit, MI.) For mixed leucocyte cultures (MLC) $10^6$ irradiated allogeneic spleen cells were added in 0·1 ml RPMI. Cultures were incubated in a 5% $CO_2$ atmosphere for 72 h. Synthesis of DNA was measured by the incorporation of tritiated thymidine ($^3$H-dT) during the last 24 h.

Each experiment with tumour-bearing and age-matched normal control cultures included multiple levels of antigen, but these dose–response titrations are not included in all the tables in this paper. Fig. 1 illustrates a typical response of normal and tumour-bearing spleen cells to a homologous tumour antigen. Data are presented as mean counts per minute (ct/min) ± s.e. for 4 replicates. With increasing amounts of antigen, $^3$H-dT incorporation increases to a peak response, and is inhibited at higher concentrations. Responses in tumour-bearing spleen cells are detected as early as 1·5 weeks after tumour inoculation, the magnitude usually increasing until the death of the animals at about 4 weeks. Spleen cells from normal animals are not stimulated.

Soluble antigens from most but not all syngeneic (non-homologous) MCA-induced tumours also stimulate tumour-bearing spleen and LNC *in vitro*, as previously reported (Forbes *et al.*, 1975; Calderwood, 1977; Calderwood *et al.*, 1977). Dose–response relationships are identical to those illustrated

FIG. 2.—Proliferative responses of spleen cells from normal (○) and tumour-bearing (●) mice to soluble syngeneic tumour antigen. Spleens from 3–7 mice were pooled and assayed 3 weeks after the inoculation of tumour cells.

here for homologous antigens. Examples of *in vitro* cross-reactions between PC1, PC5, and PC8 antigens are illustrated in Fig. 2. These data are from tumours tested *in vivo* in experiments to be described. No significant response is detected to solubilized normal muscle prepared in the same manner (Calderwood, 1977, p. 61).

Note that the background level of $^3$H-dT incorporation in tumour-bearing lymphoid cell populations is higher than that of normal cell populations, a consistent reflection of the tumour-associated lymphoid hyperplasia characteristic of these tumours (Konda *et al.*, 1973). A high degree of correlation had previously been shown between "spontaneous" and antigen-induced $^3$H-dT incorporation in tumour-bearing cell populations (Forbes *et al.*, 1975). For brevity, mean spontaneous incorporation was subtracted from mean antigen-induced levels, and results are presented as *net* ct/min for the *peak* response level of each dose–response titration.

*Subpopulation enrichment techniques.*—Complement-mediated cell lysis: Rabbit anti-mouse brain (RAMB) serum was prepared by the method of Golub (1971), absorbed once or twice with 1 part washed mouse RBC to 2 parts serum, and used at a final dilution of 1:30. Goat anti-mouse Ig (anti-Ig), generously provided by Dr Rebecca Blackstock, was used at a final dilution of 1:15. The same pool of guinea-pig complement (C′) obtained lyophilized from Microbiological Associates, Bethesda, M.D., and reconstituted to original serum volume with PRMI, was used in all experiments at a final dilution of 1:30. Two $\times 10^8$ spleen or LNC were incubated for 60 min at 37°C with the appropriate dilutions of RAMB or anti-Ig and C′ in a final volume of 30 ml RPMI, then washed $\times 3$ to remove dead cells. Null cells were prepared by sequential treatment with anti-Ig + C′ then RAMB + C′. Surviving cells were counted and used.

Control experiments established the effectiveness and specificity of both goat anti-Ig and RAMB treatments. In standard cytotoxicity tests anti-Ig + C′, diluted 1:15, consistently killed 30–40% normal spleen cells and <5% normal thymocytes. Absorbed RAMB, diluted 1:30, killed 30–40% normal spleen cells, 60–70% LNC, and 95–100% thymocytes. These cytotoxicity values are consistent with published data. Additive toxicity experiments demonstrated that RAMB and the more conventional AKR anti-Thy 1.1 killed the same set of cells (Calderwood, 1977, p. 53).

The functional efficacy of complement-mediated cell lysis was tested in each experiment by assaying the responses of the residual cell subpopulation to the B- and T-cell mitogens, LPS and PHA. Anti-Ig + C′ treatment greatly increased $^3$H-dT incorporation by surviving cells in response to PHA; it reduced to low levels or eliminated responses to LPS. RAMB + C′ treatment eliminated the PHA response of the surviving cells and increased that to LPS. The resultant data are included as examples of the results from each separation technique used.

Nylon-wool column separation: T-enriched spleen-cell fractions were eluted from nylon-wool columns prepared in 12ml disposable syringe barrels by the method of Julius *et al.* (1973). B-enriched fractions were harvested by rinsing the columns rapidly with warm medium, and then compressing the nylon wool with the syringe plunger. Collected cell fractions were pooled. The efficacy of this method of cell separation was assessed in each individual experiment in terms of function of the surviving cells, as above.

Anti-mouse immunoglobulin columns: The IgG fraction of anti-Ig was coupled to cyanogen bromide-activated Sephadex G-200, prepared as columns and used to remove B cells by the method of Chess *et al.* (1974). The purity of T-enriched subpopulations eluted by this method was tested as above.

*Modified Winn assay.*—A modification of the Winn procedure (1961) was used to test specific anti-tumour activity of lymphoid tissues or their subpopulations. Lymphocytes from spleen, lymph nodes, or lymphoid subpopulations from these organs were mixed in various ratios with freshly trypsinized washed tumour-cell suspensions and injected in a volume of 0·3 ml into one hind footpad of syngeneic mice which had been sublethally irradiated with 4 Gy delivered from an 800Ci $^{137}$Cs source at a rate of 10 Gy/min (Model M Gammator, Radiation Machinery Corporation, Parsippany, N.J.). The dorsoventral thicknesses of the footpads were measured with a Starrett Dial Gauge Micrometer (The L.S. Starrett Co., Athol, Mass.). Results are expressed as mean tumour-bearing footpad thickness $\pm$ s.e., or as mean net difference between normal and tumour-bearing foot, $\pm$ s.e.

FIG. 3.—Proliferative responses to PC8[S] antigen by surviving spleen-cell subpopulations prepared by treatment with anti-sera and C′. Spleens from 6–7 mice were pooled, treated, and assayed 1–4 weeks after the inoculation of $10^4$ PC8 cells. Net ct/min = antigen-stimulated ct/min − spontaneous ct/min. Spontaneous $H^3$-dT incorporation of the untreated tumour-bearing spleen cells was 3649, 3004, 3005, and 12,801 ct/min at Weeks 1, 2, 3, 4 respectively. Functional purity of the subpopulations was established each week by mitogen stimulation. Example: at 4 weeks, anti-Ig treatment enhanced the net PHA response of tumour-bearing spleen cells from 71,418 to 86,001 ct/min, while reducing the LPS response from 112,526 to 7638. RAMB treatment reduced the PHA responses from 71,418 to 6018 ct/min, and increased the LPS response from 112,526 to 182,543. Control data from Weeks 1, 2, and 3 were similar. ○ = untreated tumour-bearing spleen cells; △ = spleen cells treated with anti-Ig+C′ (T cells); ▲ = spleen cells treated with RAMB+C′ (B cells).

### RESULTS

### *Identification of tumour-antigen-responsive spleen and LNC subpopulations*

To determine which cells were responding to soluble antigens *in vitro*, normal and tumour-bearing spleen or LNC suspensions were separated into subpopulations by 3 different methods: complement-mediated cytolysis, nylon-wool columns and anti-Ig columns. Each method gave similar results.

First, spleen cells taken from tumour-bearing and control animals at weekly intervals over a 4-week period of tumour growth were separated into T- and B-enriched fractions by C′-mediated lysis. Surviving cells were cultured with homologous tumour antigen (Fig. 3). By the beginning of the 2nd week of tumour bearing, when the tumour mass was just palpable, whole-spleen cells responded, and the T-enriched fraction response was the greatest at every level of tumour antigen tested. T-depleted fractions were minimally responsive. At 3 weeks, the T-cell fraction was still active, but the B-enriched fraction was most active. By 4 weeks after tumour inoculation, both T-cell and B-cell fractions were highly responsive.

Spleen cells from tumour-bearing mice were also depleted of glass-wool-adherent cells, further separated into T- and B-enriched fractions, and an additional subpopulation which survived both anti-Ig or RAMB treatment (null cells). (In normal mice this null-cell subpopulation constituted less than 5% of spleen cells. As tumour growth progressed, however, the subpopulation increased, up to 25% of the spleen cells.) Table I demonstrates that 17 days after inoculation of tumour cells the T-enriched subpopulations were again the most prominent responders. By 24 days, however, responses to the T-, B- and null-cell subpopulations were equal.

Lymph-node cells collected during a similar course of tumour bearing were fractionated by the same method. Table II shows data from a typical experiment in which regional LNC were assayed 19 days after tumour inoculation. Regional LNC responses were maximal at 2–2·5 weeks and, as is shown, the proliferative response was primarily in the T-enriched subpopulation. LNC (regional, and pooled regional

TABLE I.—*Proliferative responses of spleen-cell subpopulations purified by removal of adherent cells, and then preselected by complement-mediated cytolysis*

| Spleen-cell subpopulation selected* | Peak $^3$H-dT incorporation in net ct/min/5 × 10$^5$ cells† | | | |
|---|---|---|---|---|
| | PC1[S] added | | Mitogen added (17-day data only) | |
| | 17 days | 24 days | PHA | LPS |
| *Normal* | | | | |
| Untreated | 500 | 898 | 47467 | 23431 |
| Anti-Ig + C′ (T cells) | 2073 | 734 | 105405 | 7921 |
| RAMB + C′ (B cells) | 1130 | 703 | −81 | 82063 |
| Anti-Ig + RAMB + C′ (null cells) | 3274 | 2262 | 245 | 20266 |
| *PC1-bearing* | | | | |
| Untreated | 21505 | 22841 | 9911 | 40471 |
| Anti-Ig + C′ (T cells) | 22959 | 21607 | 23524 | 2469 |
| RAMB + C′ (B cells) | 12159 | 27115 | 6026 | 53745 |
| Anti-Ig + RAMB + C′ (null cells) | 8919 | 20930 | 4309 | −4012 |

* Spleen cells from 12 normal or 9 tumour-bearing animals were pooled. Adherent cells were removed by passing the cell suspensions twice through glass wool before treatment with anti-Ig + C′, RAMB + C′, or sequential treatments with both antisera, and then tested for proliferative responses to mitogens or to PC1[S] antigen.

† Net ct/min = peak antigen-stimulated ct/min − spontaneous ct/min/5 × 10$^5$ cells.

Mitogen responses for surviving normal and tumour-bearing spleen-cell subpopulations did not differ significantly at 24 days.

TABLE II.—*Proliferative responses of regional lymph-node cell subpopulations preselected by complement-mediated cytolysis*

| Lymph-node cell subpopulations selected* | Peak $^3$H-dT incorporation in net ct/min/10$^6$ cells | | |
|---|---|---|---|
| | Antigen or mitogen added to culture | | |
| | PC1[S] | PHA | LPS |
| *Normal* | | | |
| Untreated | 14 | 69575 | 8660 |
| Anti-Ig + C′ (T cells) | −24 | 87341 | 5803 |
| RAMB + C′ (B cells) | 3339 | 24574 | 39317 |
| *PC1-bearing* | | | |
| Untreated | 46277 | 86255 | 37007 |
| Anti-Ig + C′ (T cells) | 15430 | 111031 | 15973 |
| RAMB + C′ (B cells) | −67 | −1709 | 89689 |

* LNC from 30 normal or tumour-bearing mice were pooled before treatment with the indicated antisera + C′. Regional LNC from the inguinal and paraortic nodes draining the tumour site were collected 19 days after the i.m. inoculation of PC1.

and non-regional) did not respond detectably to soluble tumour antigen after about 3 weeks of tumour bearing.

To confirm the early dominance of the T-cell subpopulations enriched by C′-mediated lysis, other methods of separation were used. The data shown in Table III illustrate the findings in fractions obtained by passage through anti-Ig columns. Spleen-cell responses to tumour antigen at 2 weeks were not reduced by removal of the immunoglobulin-bearing cells. The same cell subpopulation retained responses to PHA and to alloantigens, but lost reactivity to LPS.

Fractionation by nylon-wool columns gave similar data, as illustrated in Table IV. By the 2nd week of tumour bearing, when proliferative responses to tumour antigen had become highly significant, removal of T cells reduced the responses to both the homologous tumour antigen PC1[S] and to cross-reacting antigen PC5[S]. By the 3rd week, both T- and B- cell responses were vigorous. These data support those derived from the other fractionation techniques, and those derived using the other tumours.

*Correlation between subpopulation response* in vitro, *and capacity to inhibit tumour growth* in vivo

It became clear, in the types of experiments described, that tumour-specific proliferative responses occurred chiefly in T-cell subpopulations during early tumour

TABLE III.—*Proliferative responses of spleen cells passed through anti-mouse Ig columns*

| Spleen-cell subpopulations* | Peak $^{3}$H-dT incorporation in net ct/min/$10^{6}$ spleen cells — Antigen or mitogen added to culture: PC5[S] | PHA | LPS | C57BL/6 | CBA |
|---|---|---|---|---|---|
| *Normal* | | | | | |
| Unfractionated | 5429 | 29664 | 34674 | −1250 | 21924 |
| Passed column (T cells) | 5670 | 117189 | 23240 | −964 | 27884 |
| *PC5-bearing* | | | | | |
| Unfractionated | 20083 | 6142 | 11046 | −1317 | 11211 |
| Passed column (T cells) | 21245 | 21459 | 2437 | −1162 | 9526 |

* Spleen cells of 5 normal or 3 tumour-bearing mice were pooled and passed through anti-Ig columns. Cells which were not retained by the column were tested for tumour-specific responses and functional responses to mitogens and irradiated syngeneic (C57BL) or allogeneic [CBA] spleen cells.

TABLE IV.—*Proliferative responses of spleen-cell subpopulations passed through nylon-wool columns*

Peak $^{3}$H-dT incorporation in net ct/min/$10^{6}$ spleen cells

| Spleen-cell subpopulation selected* | PC1[S] added | | PC5[S] added | | Mitogen added (Wk 3 only) | |
|---|---|---|---|---|---|---|
| | Wk 2 | Wk 3 | Wk 2 | Wk 3 | PHA | LPS |
| *Normal* | | | | | | |
| Unfractionated | 846 | −317 | 1691 | 335 | 71682 | 87927 |
| Nylon passed (T cells) | 1010 | 926 | 2441 | 2243 | 72817 | 6400 |
| Nylon retained | 259 | −192 | 494 | 226 | 50499 | 90552 |
| *PC1-bearing* | | | | | | |
| Unfractionated | 3939 | 16900 | 2451 | 14524 | 28621 | 98238 |
| Nylon passed (T cells) | 4583 | 16200 | 8590 | 22959 | 58000 | 32597 |
| Nylon retained | 2701† | 17069 | 1940 | 14393 | 15680 | 120743 |

* Spleen cells from 5 normal or tumour-bearing mice were pooled prior to passage through nylon-wool columns. Control mitogen responses for Week 2 were similar.

† Significantly below the response of unfractionated PC1 spleen cells ($P < 0{\cdot}001$).

growth, but were strong also in B-enriched and null cells after 3–4 weeks of tumour bearing. It was of interest to determine whether the *in vitro* behaviour of such subpopulations was associated with a capacity to retard tumour growth *in vivo*. Lymphocyte subpopulations were tested simultaneously for *in vitro* response to soluble tumour antigen and for *in vivo* capacity to inhibit tumour growth in an adoptive host.

The results of a representative experiment are shown in Table V. In this experiment spleen cells were taken at a time after tumour inoculation (2·5 weeks) when T-, B- and null-cell subpopulations all responded vigorously *in vitro* to tumour antigen. The spleen cells were fractionated by C′-mediated lysis, and surviving cell subpopulations were tested with homologous antigen. Aliquots of the same subpopulations, mixed with viable homologous tumour cells at a 2000:1 ratio, were inoculated into the right hind footpads of groups of 5 sublethally irradiated mice. Footpad measurements 22 days after inoculation showed that only the T-cell subpopulations inhibited tumour growth. At this time, most animals from the T-cell group were still tumour-free. Unfractionated tumour-bearing spleen cells retarded tumour growth slightly. Control cells were ineffective.

Similar experiments demonstrated that tumour-bearing lymphocytes best retarded tumour growth in adoptive hosts if taken 1·5–2·5 weeks after tumour inoculation, when the proliferation by the T-

TABLE V.—*Comparison of* in vitro *proliferative responses to tumour antigen with* in vivo *inhibition of tumour growth*

| Spleen-cell subpopulation selected* | Peak $^{3}$H-dT incorporation† Antigen or mitogen added PC1[S] | PHA | LPS | Winn assay data FPS‡ | P§ |
|---|---|---|---|---|---|
| *Normal* | | | | | |
| Untreated | 987 | 59249 | 16579 | 4·80 ± 0·18 | — |
| Anti-Ig + C′ (T cells) | 1918 | 111642 | 4116 | 4·34 ± 0·26 | NS |
| RAMB + C′ (B cells) | 2180 | 443 | 37071 | 5·50 ± 0·31 | NS |
| Anti-Ig + RAMB + C′ (null cells) | 3419 | 8862 | 3678 | 4·05 ± 0·08 | NS |
| *PC1-bearing* | | | | | |
| Untreated | 11156 | 18227 | 26269 | 2·83 ± 0·52 | 0·01 |
| Anti-Ig + C′ (T cells) | 17387 | 38293 | −4056 | 1·98 ± 0·13 | 0·001 |
| RAMB + C′ (B cells) | 20017 | 16495 | 6975 | 4·77 ± 0·12 | NS |
| Anti-Ig + RAMB + C′ (null cells) | 20996 | 15760 | −2015 | 3·58 ± 0·34 | NS |

* Spleen cells from 10 normal or 10 tumour-bearing animals inoculated 17 days earlier with PC1 were pooled before treatment with antisera + C′.

† Net ct/min/$5 \times 10^{5}$ surviving cells.

‡ Footpad swelling = mean dorsoventral thickness in mm ± s.e. of the right hind footpad, which had been inoculated 22 days earlier with a mixture of $5 \times 10^{2}$ viable PC1 cells mixed $2 \times 10^{6}$ viable selected or normal spleen cells. Donor lymphocytes were collected after 17 days bearing PC1. Mean normal left footpad measurement was 1·78 ± 0·00 mm, and has not been subtracted.

§ All footpad measurements are compared with those of mice receiving the mixture of tumour cells and untreated normal spleen cells. NS = no significant difference.

FIG. 4.—Growth curves of PC1 tumour inoculated together with regional LNC of normal or PC1-bearing mice. $10^{3}$ PC1 tumour cells were inoculated together with $10^{6}$ T-enriched LNC into the right hind footpad (FP) of sublethally irradiated mice. Normal or tumour-bearing regional LNC were pooled from groups of 10 mice after 1, 2, or 3 weeks tumour bearing. Enrichment was by complement-mediated cytolysis. Net FP = mean mm difference in footpad thickness between tumour-bearing and normal feet (groups of 5 mice). 0 = normal LNC + PC1 cells; ● = tumour-bearing regional LNC + PC1 cells.

enriched fraction was most evident in the *in vitro* assay. For example, Fig. 4 illustrates growth curves from experiments in which PC1 tumour cells were mixed with normal LN T cells or LN T cells collected from tumour-bearing mice after 1, 2 or 3 weeks. Inhibition of tumour growth by tumour-bearing LN T cells was evident at 2 weeks of tumour bearing, correlating closely with the time of greatest *in vitro*

reactivity. Similar results were obtained in experiments using spleen cells after 1, 2 and 3 weeks tumour bearing.

The lymphoid cell: tumour cell ratio used in the above experiments was 1000–2000:1. Spleen or LNC were found to exhibit similar anti-tumour activity at lymphoid-cell: tumour-cell ratios between

FIG. 5.—Growth curves of tumours inoculated together with various ratios of spleen T cells. The right hind footpads of sublethally irradiated mice were inoculated with $10^3$ PC1 cells and $10^4$, $10^5$, $10^6$, or $10^7$ T cells (lymphoid: tumour cell ratios of $10–10^4$: 1) from spleens of normal or PC1-bearing mice. T cells were prepared by anti-Ig+C′ treatment of spleen cells pooled from 10 mice, and surviving cells were used. Net FP as in Fig. 4. ○ = normal spleen T cells + PC1 cells; ● = tumour-bearing spleen T cells collected 2 weeks after tumour inoculation, = PC1 cells.

FIG. 6.—Growth curves of tumours inoculated together with normal, homologous, or syngeneic (non-homologous) regional LNC. The left hind footpads of sublethally irradiated mice were injected with $10^3$ PC8 or PC5 tumour cells and $10^6$ regional LNC from either age-matched normal or tumour-bearing mice. Pooled LNC were collected after 1, 2, and 3 weeks of tumour bearing from groups of 10–15 mice. ○ = normal LNC + tumour cells; ● = PC1-bearing regional LNC + tumour cells; □ = PC5-bearing regional LNC + tumour cells; ■ = PC8-bearing regional LNC + tumour cells.

FIG. 7.—Growth curves of tumours inoculated together with normal, homologous, or syngeneic (non-homologous) regional LN T cells. The left hind footpads of sublethally irradiated mice were injected with $10^3$ PC1 or PC8 tumour cells and $10^6$ regional LN T cells from either age-matched normal or tumour-bearing mice. Pooled regional LNC were collected 2·5 weeks after tumour inoculation from groups of 15 mice. T cells were prepared by complement-mediated cytolysis. ○ = normal LN T cells + tumour cells; ● = PC1-bearing regional LN T cells + tumour cells; ■ = PC8 bearing regional LN T cells + tumour cells.

$10^2$:1 and $10^4$:1, when taken 1–3 weeks after tumour inoculation (Fig. 5). Since the response to soluble antigens from syngeneic tumours was shared or cross-reactive in the *in vitro* assay (Forbes *et al.*, 1975; Fig. 2, Table IV), specificity of inhibition of tumour growth was also examined in the modified Winn assay. Cross-reactive anti-tumour activity was not demonstrated in any combination of syngeneic tumour examined. Inhibition was limited to the homologous tumour when tested at a ratio of 1000:1. For example, Fig. 6 shows growth curves of PC8 and PC5 tumour injected together with regional LNC taken from animals bearing either the homologous tumour or syngeneic tumours, after 1, 2 or 3 weeks of tumour bearing. Only LNC regional to the homologous tumour retarded tumour growth, and this activity was maximal at 2 weeks, as before. In the experiment shown in Fig. 7, T cells from normal lymph nodes or lymph nodes regional to PC8 or PC1 tumour were collected after 2·5 weeks of tumour bearing and inoculated with PC1 or PC8. Only homologous regional LN T cells inhibited tumour growth. All 3 tumours showed shared or cross-reactive responses in the *in vitro* assay (Fig. 2). In no combination of syngeneic tumours tested was adoptive inhibition of tumour growth detectable.

### DISCUSSION

The experiments reported here affirm that the lymphoid-cell response to soluble tumour antigen in the *in vitro* proliferation assay reflects a heterogeneous population which changes during the course of tumour growth. Early responses are largely T-cell-mediated and are detected first in regional lymph nodes, later in other lymph nodes and spleen. B-cell proliferation in response to soluble antigen follows shortly in both lymph-node and spleen subpopulations. Null-cell responses are most prominent late in the course of tumour bearing. Although all lymphoid cell subpopulations were responsive to soluble tumour antigen *in vitro*, only the T-enriched subpopulation inhibited tumour growth appreciably in adoptive hosts. This anti-tumour activity was maximal during the peak of T-cell activity *in vitro*, about 2 weeks after tumour inoculation. Neither B cells nor null cells, as defined herein, detectably retarded tumour growth at any time, despite the vigorous antigen-induced proliferative responses observed late in tumour bearing. Tumour inhibition appeared to be correlated with tumour-specific transplantation antigen (TSTA) activity (as defined by *in vivo* immunization techniques), whereas the *in vitro* proliferation assay identified shared antigens, even in the T-enriched subpopulations.

The cell separation techniques used here made possible the isolation of lymphoid cell subpopulations at the peak of their *in vitro* responsiveness to tumour-associated antigens. Even though no single technique of T- or B-cell purification yields subpopulations completely free from other cell classes or macrophages, the data from all 3 enrichment techniques used here were totally congruent. Operationally, preselection by C′-mediated lysis provided the most convenient and effective method of deriving relatively pure subpopulations, as judged by functional responses to mitogens.

The importance of T cells to tumour rejection in other tumour systems is well known (Kearney *et al.*, 1975; Bernstein *et al.*, 1976). T-cell inhibition of homologous tumour growth in adoptive hosts, shown here for chemically induced sarcomas, occurs early in tumour bearing, when the *in vitro* proliferative responses are chiefly T-cell mediated. This is consistent with the proposition that early T-cell proliferation, as revealed in the *in vitro* assay, reflects the generation of a subset of tumour-specific T cells directed toward TSTA, as well as subsets which are cross-reactive with, or share antigens with, syngeneic tumours.

Old *et al.* (1962) found that peritoneal-exudate cells (PEC) gave singular inhibition of tumour growth in the adoptive host, but LN or spleen cells from the same hosts had no effect. Their experiments did not involve purified cell subpopulations, or explore the early phase of tumour bearing described here.

We have also found (Smith *et al.*, 1978; Chauvenet & Smith, 1979) that a high degree of growth inhibition is afforded by peritoneal-exudate cells in the adoptive-transfer technique described here. Such inhibition was clearly T-cell mediated. Moreover, long-term-cloned T-cell lines which show cytotoxicity *in vitro* and growth inhibition *in vivo* for homologous tumour can be derived from the same PEC populations (Smith *et al.*, 1980).

The data reported here, taken with earlier studies, suggest strongly that tumour-specific T cells are generated early by proliferation in response to tumour presence, and that T-cell-mediated inhibition of growth of chemically induced sarcomas is primarily directed toward an antigenic system or systems not shared with other syngeneic tumours. A corollary of this conclusion is that antigen-responsive T cells not protective *in vivo* are generated at the same time, and that these subsets respond to specificities shared with syngeneic tumours. These experiments emphasize also that tumour-antigen-associated proliferation stimulated by chemically induced sarcomas also occurs in B-enriched subpopulations and a large subpopulation of null cells, chiefly occurring later in tumour bearing. Neither of these subpopulations inhibits tumour growth in an adoptive host.

This work was supported in part by National Institutes of Health Grants HD-00384, CA-15334, CA-09126, CB-53933, and gifts to the Mary and Ryan Whisenant Cancer Research Fund.

### REFERENCES

ADLER, W. H., TAKAGUCHI, T., MARCH, B. & SMITH, R. T. (1970*a*) Cellular recognition by mouse lymphocytes *in vitro*. I. Definition of a new technique and results of stimulation by PHA and specific antigens. *J. Exp. Med.*, **131**, 1049.

ADLER, W. H., TAKAGUCHI, T., MARCH, B. & SMITH, R. T. (1970*b*) Cellular recognition by mouse lymphocytes *in vitro*. II. Specific stimulation by histocompatibility antigens in mixed cell cultures. *J. Immunol.*, **105**, 984.

BERNSTEIN, I. D., WRIGHT, P. W. & COHEN, E. (1976) Generation of cytotoxic lymphocytes *in vitro*: Response of immune rat spleen cells to a syngeneic Gross virus-induced lymphoma in mixed lymphocyte-tumor culture. *J. Immunol.* **116**, 1367.

CALDERWOOD, M. B. (1977) *Cell-Mediated Immunity to Tumor-Associated Antigens*. Dissertation, University of Florida.

CALDERWOOD, M. B., FORBES, J. T. & SMITH, R. T. (1977) Soluble tumor antigen-induced lymphocyte proliferation: Effects of serum from normal and tumor-bearing mice. *Int. J. Cancer*, **20**, 400.

CHAUVENET, P. H. & SMITH, R. T. (1979) Demonstration *in vitro* of cytotoxic T-cells with apparent specificity toward tumor-specific transplantation antigens on chemically induced tumor. *J. Immunol.*, **123**, 2575.

CHESS, L., MACDERMOTT, R. P. & SCHLOSSMAN, S. F. (1974) Immunologic function of isolated human lymphocyte subpopulations. I. Quantitative isolation of human T and B cells and response to mitogens. *J. Immunol.*, **113**, 1113.

FORBES, J. T., NAKAO, Y. & SMITH, R. T. (1975) Tumor-specific immunity to chemically induced tumors. Evidence for immunologic specificity and shared antigenicity in lymphocyte response to soluble tumor antigens. *J. Exp. Med.*, **141**, 1181.

GAINOR, B. J., FORBES, J. T., ENNEKING, W. F. & SMITH, R. T. (1976) Specific antigen stimulated lymphocyte proliferation in osteosarcomas. *Cancer*, **37**, 743.

GOLUB, E. S. (1971) Brain-associated θ antigen: Reactivity of rabbit anti-mouse brain with mouse lymphoid cells. *Cell. Immunol.*, **2**, 353.

GUTTERMAN, J. U., MAVLIGIT, G. M. & MCCREDIE, K. B. (1972) Antigen solubilized from human leukemia: Lymphocyte stimulation. *Science*, **177**, 1114.

JEHN, U. W., NATHANSON, L., SCHWARTZ, R. S. & SKINNER, M. (1970) *In vitro* lymphocyte stimulation by a soluble antigen from malignant melanoma. *N. Engl. J. Med.*, **283**, 329.

JULIUS, M. H., SIMPSON, E. & HERZENBERG, L. A. (1973) A rapid method for the isolation of functional thymus-derived murine lymphocytes. *Eur. J. Immunol.*, **3**, 645.

KEARNEY, R., BASTEN, A. & NELSON, D. S. (1975) Cellular basis for the immune response to methylcholanthrene induced tumors in mice. *Int. J. Cancer*, **15**, 438.

KONDA, S., NAKAO, Y. & SMITH, R. T. (1973) The stimulatory effect of tumor bearing upon the T- and B-cell subpopulations of the mouse spleen. *Cancer Res.*, **33**, 2247.

MAVLIGIT, G. M., AMBUS, U., GUTTERMAN, J. U., HERSCH, E. M. & MCBRIDE, C. M. (1973) Antigen solubilized from human solid tumors: Lymphocyte stimulation and cutaneous delayed hypersensitivity. *Nature* (*New Biol.*), **243**, 188.

MELTZER, M. S., OPPENHEIM, J. J., LITTMAN, B. H., LEONARD, E. J. & RAPP, H. J. (1972) Cell-mediated tumor immunity measured *in vitro* and *in vivo* with soluble tumor-specific antigens. *J. Natl Cancer Inst.*, **49**, 727.

OLD, L. H., BOYSE, E. A., CLARKE, D. A. & CARSWELL, E. A. (1962) Antigenic properties of chemically induced tumors. *Ann. N.Y. Acad. Sci.*, **101**, 80.

REISFELD, R. A., PELLEGRINO, M. A. & KAHAN, B. D. (1971) Salt extraction of soluble HL-A antigens. *Science*, **172**, 1134.

SMITH, R. T. (1975) Tumor antigen as the controlling element in the tumor–host relationship. In *Immunobiology of the Tumor–Host Relationship*, Eds Smith & Landy. New York: Academic Press, Inc. p. 12.

SMITH, R. T., CHAUVENET, P. H., CALDERWOOD, M. & MCARTHUR, C. (1978) Correlation of *in vitro* anti-TSTA activity with tumor growth retardation in adoptive hosts. *Proc. IX Ann. Meeting Scand. Soc. Immunol., Stockholm.* p. 85.

SMITH, R. T., KONAKA, Y., CALDERWOOD, M., SHIMIZU, S., SMITH, L. & KLEIN, P. A. (1980) Specific immune responses to chemically induced tumors: Relation between tumor specific antigens recognised by cloned T-cell lines and by antibody producing hybridomas, to tumor specific protection *in vivo*. In *Fundamental Mechanisms in Cancer Immunology*. Amsterdam: Elsevier, North-Holland. p. 32.

VANKY, F., KLEIN, E., STJERNSWARD, J. & NILSONNE, U. (1974) Cellular immunity against tumor-associated antigens in humans: Lymphocyte stimulation and skin reaction. *Int. J. Cancer*, **14**, 277.

WINN, H. J. (1961) Immune mechanisms in homotransplantation. II. Quantitative assay of the immunologic activity of lymphoid cells stimulated by tumor homografts. *J. Immunol.*, **86**, 228.